# P-1943. Outcome of invasive Candida infections in Europe and the US: Results from an ongoing multinational study [2024-2026]

**DOI:** 10.1093/ofid/ofaf695.2111

**Published:** 2026-01-11

**Authors:** Laman Rahimli, Nijat Azimli, Natalia Vasenda, Jon Salmanton-Garcia, Rosanne Sprute, Iker Falces-Romero, Maricela Valerio, Ana Soriano, Andrea Gutierrez Villanueva, Patricia Monzo-Gallo, Carolina Garcia-Vidal, Frank Hanses, Jose A Vazquez, Patrick Yu, Ulrike Scharmann, Oliver A Cornely, Danila Seidel

**Affiliations:** University of Cologne, Faculty of Medicine and University Hospital Cologne, Department I of Internal Medicine, Excellence Center for Medical Mycology (ECMM), Cologne, NRW, Germany; University of Cologne, Faculty of Medicine and University Hospital Cologne, Institute of Translational Research, Cologne Excellence Cluster on Cellular Stress Responses in Aging-Associated Diseases (CECAD), Cologne, NRW, Germany, Cologne, Nordrhein-Westfalen, Germany; University Hospital Cologne, Cologne, Nordrhein-Westfalen, Germany; University of Cologne, Faculty of Medicine and University Hospital Cologne, Department I of Internal Medicine, Excellence Center for Medical Mycology (ECMM), Cologne, NRW, Germany; University of Cologne, Faculty of Medicine and University Hospital Cologne, Institute of Translational Research, Cologne Excellence Cluster on Cellular Stress Responses in Aging-Associated Diseases (CECAD), Cologne, NRW, Germany, Cologne, Nordrhein-Westfalen, Germany; University Hospital Cologne, Cologne, Germany, Cologne, Nordrhein-Westfalen, Germany; University Hospital Cologne, Cologne, Nordrhein-Westfalen, Germany; Hospital Universitario La Paz, Microbiology and Parasitology, Madrid, Madrid, Madrid, Spain; Hospital General Universitario Gregorio Marañón. Clinical Microbiology and Infectious Diseases Department. Instituto de Investigación Sanitaria Gregorio Marañón, Madrid, Madrid, Spain; Hospital General Universitario Gregorio Marañón, Madrid, Madrid, Spain; Hospital Puerta de Hierro Majadahonda, Madrid, Madrid, Spain; Hospital Clinic of Barcelona, Barcelona, Catalonia, Spain; Hospital Clinic, Department of Hematology, Barcelona, Spain, Barcelona, Catalonia, Spain; Abteilung für Krankenhaushygiene und Infektiologie, Universitätsklnikum Regensburg, Regensburg, Bayern, Germany; Medical College of Georgia at Augusta University, Augusta, GA; Medical College of Georgia, ugusta University, Augusta, Georgia; University of Duisburg-Essen, Essen, Nordrhein-Westfalen, Germany; University of Cologne, Faculty of Medicine and University Hospital Cologne, Cologne, Nordrhein-Westfalen, Germany; University Hospital of Cologne, Cologne, Nordrhein-Westfalen, Germany

## Abstract

**Background:**

Invasive *Candida* infection cause significant morbidity and mortality, especially in immunocompromised patients. The FungiScope Candida Campaign 2024–2026 aims to analyze clinical data to better understand risk factors, treatments, and outcomes in this population across different geographical regions.

**Methods:**

Anonymized patient data were collected via an online questionnaire (www.clinicalsurveys.net for Europe, Carelane for the USA), capturing demographics, clinical history, diagnostic findings, antifungal treatment, source control measures, and patient outcomes.

**Results:**

A total of 463 patients with invasive *Candida* infection were included by April 2025, with a median age of 67 years (range: 18–102); 62.2% were male. Most cases were reported from Germany (n=213; 46.0%), followed by Spain (171; 36.9%) and Italy (39; 8.4%).

Frequent risk factors included central venous catheter use (54.6%), ICU treatment (42.8%), chronic cardiovascular disease (40.6%), hematological/oncological conditions (38.7%), and recent major surgery (35.9%). Chronic kidney diseases/acute kidney injury and uncontrolled diabetes were present in 22.9% and 22.5% of cases, respectively.

Most patients received antifungal therapy (92%; median 14 days, range 1–306). First-line agents included caspofungin (43.6%), anidulafungin (23.7%), and fluconazole (21.1%), while fluconazole (47.3%) and caspofungin (18.5%) predominated as second-line treatment.

Germany predominantly used caspofungin (75.1%), whereas Spain favored anidulafungin (51.2%) as the first-line agent. In Italy, fluconazole (35.9%) and caspofungin (33.3%) were the leading choices. Survival was not significantly influenced by either the initial antifungal therapy (χ²(9) = 13.76, p = 0.131) or the Candida species (albicans vs. non-albicans; χ²(1) = 0.54, p = 0.462).Overall mortality was 39.4%.

**Conclusion:**

In this multinational cohort, echinocandins were predominantly used as first-line treatment and fluconazole for second-line. Neither initial antifungal choice nor *Candida* species (*albicans* vs. non-*albicans*) significantly impacted survival, highlighting the urgent need for optimized management strategies to reduce high mortality rates.

**Disclosures:**

Jon Salmanton-Garcia, MSc, MPH, PhD, menarini, gilead, astrazeneca, pfizer: Honoraria Rosanne Sprute, Dr., Hikma: Honoraria|Mundipharma: Honoraria|Pfizer: Honoraria Oliver A. Cornely, Prof. Dr., Al-Jazeera Pharmaceuticals/Hikma: Honoraria|Basilea: Advisor/Consultant|Cidara: Advisor/Consultant|Cidara: Board Member|Cidara: Grant/Research Support|Elion: Advisor/Consultant|F2G: Grant/Research Support|Gilead: Advisor/Consultant|Gilead: Grant/Research Support|Gilead: Honoraria|GlaxoSmithKline: Advisor/Consultant|GlaxoSmithKline: Honoraria|Grupo Biotoscana/United Medical/Knight: Honoraria|Melinta: Advisor/Consultant|Melinta: Board Member|MSD: Honoraria|Mundipharma: Advisor/Consultant|Mundipharma: Grant/Research Support|Mundipharma: Honoraria|Pfizer: Advisor/Consultant|Pfizer: Grant/Research Support|Pfizer: Honoraria|Pulmocide: Board Member|Scynexis: Advisor/Consultant|Scynexis: Grant/Research Support|Shionogi: Advisor/Consultant|Shionogi: Honoraria

